# Hybrid Deep Learning Framework for Melanoma Diagnosis Using Dermoscopic Medical Images

**DOI:** 10.3390/diagnostics14192242

**Published:** 2024-10-08

**Authors:** Muhammad Mateen, Shaukat Hayat, Fizzah Arshad, Yeong-Hyeon Gu, Mugahed A. Al-antari

**Affiliations:** 1 School of Electronic and Information Engineering, Soochow University, Suzhou 215006, China; 2 Department of Software Engineering, International Islamic University, Islamabad 44000, Pakistan; s.hayat@iiu.edu.pk; 3 Department of Computer Science, Air University Multan Campus, Multan 61000, Pakistan; fizzaa995@gmail.com; 4 Department of Artificial Intelligence and Data Science, College of AI Convergence, Daeyang AI Center, Sejong University, Seoul 05006, Republic of Korea

**Keywords:** skin lesion, melanoma detection, deep learning, ultraviolet rays, lesion segmentation, classification

## Abstract

**Background:** Melanoma, or skin cancer, is a dangerous form of cancer that is the major cause of the demise of thousands of people around the world. **Methods:** In recent years, deep learning has become more popular for analyzing and detecting these medical issues. In this paper, a hybrid deep learning approach has been proposed based on U-Net for image segmentation, Inception-ResNet-v2 for feature extraction, and the Vision Transformer model with a self-attention mechanism for refining the features for early and accurate diagnosis and classification of skin cancer. Furthermore, in the proposed approach, hyperparameter tuning helps to obtain more accurate and optimized results for image classification. **Results:** Dermoscopic shots gathered by the worldwide skin imaging collaboration (ISIC2020) challenge dataset are used in the proposed research work and achieved 98.65% accuracy, 99.20% sensitivity, and 98.03% specificity, which outperforms the other existing approaches for skin cancer classification. Furthermore, the HAM10000 dataset is used for ablation studies to compare and validate the performance of the proposed approach. **Conclusions:** The achieved outcome suggests that the proposed approach would be able to serve as a valuable tool for assisting dermatologists in the early detection of melanoma.

## 1. Introduction

Cancer is a leading cause of death globally and in general. The World Health Organization (WHO) predicts that by the year 2030, cancer would have surpassed all other causes of death worldwide to claim the lives of 13.1 million people [1]. The early detection of cancer greatly increases the likelihood that therapy will be effective. Early cancer diagnoses, also known as screening and down staging, are the two main components that make up earliest cancer detection. Early detection concentrates on the detection of patients with symptoms as early as is practicable. In contrast, screening requires evaluating a healthy person to identify those who have cancers before any symptoms arise. The early detection of cancer patients is the focus of early diagnosis. The goal of programs that focus on early diagnosis is to cut down on the number of patients who receive a diagnosis at a later stage [1].

The early diagnosis, therapy, and end-of-life care are currently the focuses of cancer care. The “multinational association of supportive care in cancer” defines supportive care as “the management and prevention of adverse consequences of cancer and its medication” [2]. Reducing the time it takes from the appearance of symptoms to the start of therapy is the primary goal of early diagnosis programs, which aim to do this by ensuring that the diagnostic and treatment services are easily available, providing high-quality healthcare at a reasonable cost, and responding appropriately and quickly because of proper training and clear referral procedures.

Screening is the process of identifying individuals within a healthy population who have a disease but do not yet show any symptoms by using very straightforward diagnostic procedures. While there is evidence that improved patient outcomes can result from the early detection and treatment of skin cancer, there is also a paucity of data proving the application of widespread screening programs. Furthermore, there is a wide range in physicians’ abilities to recognize cancerous lesions, depending on their level of education and experience.

**Figure 1:**
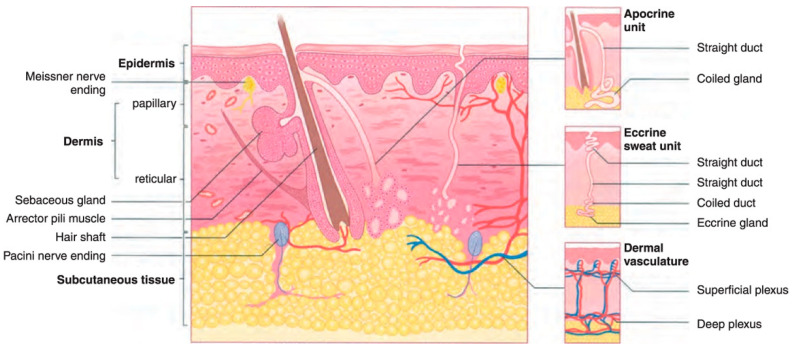
The dermis and epidermis layers of human skin made up of basal cells and squamous cells and melanocytes [5].

Among the several kinds of cancer that can develop in the United States, skin cancer is the most common type of cancer. Twenty percent of Americans will eventually acquire skin cancer in their lifetimes, according to estimates [3]. However, not every instance of skin cancer is fatal. On the other hand, saving lives depends on detection at an early stage. To better understand how skin cancer is detected and diagnosed, more studies of the human skin and its various types are required. Melanoma is the deadliest type of skin cancer since it has a propensity to extend to other bodily parts, particularly to vital organs such as the eyes, face, feet, and limbs, and very rarely to internal body locations such as the nose or mouth. The precise reason why some people get melanoma skin cancer is unknown, however, an increased likelihood of developing the disease is associated with prolonged exposure to ultraviolet rays [4,5]. Ultraviolet (UV) rays have the ability to cause harm to the cells that make up the skin [6]. This damage might result in sunburn in the short term. The damage caused by ultraviolet rays accumulates over time and can lead to changes in skin texture, an acceleration of the aging process, and even skin cancer. The most common place for melanomas to begin is within the skin (epidermis), which is the outermost layer of skin. This layer is composed of the following three types of cells: Squamous cells, which are characterized by their thinness and flatness, make up the epidermis’ topmost layer [7]. Basal cells, which are subordinate to the more prominent squamous cells, are spherical and located underneath the latter [7,8]. Melanocytes are located in the epidermis’ basal layer, where they produce melanin. The skin’s natural color comes from a substance called melanin. Darker skin is the result of an increase in melanin production [9] when skin is exposed to sunlight. The two most common types of skin cancer are squamous cell carcinoma and basal cell carcinoma. Basal cells are the starting point for the development of basal cell cancer. Cancer of the squamous cells, which make up the skin’s outermost layer, is called squamous cell carcinoma [8]. Both are usually curable although they are both disfiguring and costly to treat. Figure 1 presents the anatomy of the skin for a better understanding of the epidermis and dermis layers.

**Figure 2:**
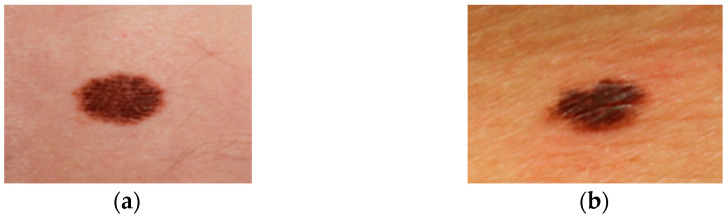
Normal moles and abnormal moles. (**a**) Normal moles/nevi/nevus, (**b**) abnormal moles/dysplastic nevus (DN), atypical melanocytic nevus (AMN).

With early detection, skin problems have a 90% chance of being cured, but with late detection, that chance drops to 50%. The development of high-resolution, non-invasive imaging methods has enhanced the precision and accuracy of detecting skin cancer or malignancies [10]. Overtreatment, one of the main causes of poor diagnostic accuracy, is treatment resulting from a mistaken negative diagnosis of melanoma or an incorrect positive diagnosis [11]. Exorbitant treatment expenses are largely attributable to false-positive diagnoses, which result in the unnecessary excision of a high volume of benign tumors for subsequent, unrestricted assessment and biopsy. Moles, both common and uncommon, are depicted in Figure 2.

The main contributions of the proposed work are given below:

- A novel AI-based framework is designed to obtain a precise and efficient melanoma diagnosis, covering a hybrid approach that integrates Inception-ResNet-v2 to extract the deep features and Vision Transformer for recognition.
- To improve the performance of the proposed framework for disease prediction, an automated system of segmentation is utilized through the U-Net architecture for inputting dermoscopic images and leveraging the advantage of residual connections of Inception-ResNet-v2 to achieve state-of-the-art results.
- The ablation study is carried out in a comprehensive way to evaluate the trustworthiness of the proposed model and to reflect the efficiency of each component within the proposed framework.

The rest of the paper is structured as follows: Section 2 presents the latest deep learning studies for skin lesion classification and segmentation. Section 3 describes the proposed methodology, while Section 4 explains the results of the proposed model and presents the discussion of the work. Section 5 covers the conclusion and future work.

## 2. Related Work

Currently, the most frequently used imaging modalities that help to diagnose skin malignancies are dermatoscopy [12], ultrasound [13], optical coherence tomography [14], reflectance confocal microscopy [15], and hyperspectral imaging [10,16]. The melanoma skin lesions cannot be identified as normal tumors using any of the skin imaging methods mentioned above. Understanding the various forms of skin cancer requires the doctors to be able to use trustworthy diagnostic techniques when diagnosing skin cancers, especially melanoma; thus, the generally accepted and repeatable ABCD-E rule is applied. Stolz et al. [11] established this approach in 1994. The ABCD-E rule says the following: A for asymmetry, B for border, C for color, D for diameter, and E for evolving by time. The most important characteristic of melanoma skin lesions, which makes them difficult to detect, is that they evolve with the passage of time, such as in size, shape, changing color, or sometime itching or bleeding. However, melanomas can be smaller than 6 mm in rare instances, in which case the ABCD-E rule does not apply. As a result, the use of AI-based melanoma skin cancer detection systems has skyrocketed for the early identification of this type of cancer and to gain the best treatment options. Artificial intelligence in diagnostic technologies has the ability to provide more people with high-quality medical care, which is a substantial potential social benefit. Initially, the custom feature-based approach was used to detect melanoma. Codella et al. [17] proposed a manually coded feature extraction for the detection of melanoma skin lesions by combining edge and color histograms with local binary patterns. In [18], Marques, Francisco, Mendonca, and Barata proposed a method for the detection of melanoma skin lesions through the combination of two algorithms that use local image elements as well as global image elements. The Laplacian pyramid and gradient histogram are used to capture the features of global image elements, such as shape, size, color, texture, etc., of the melanoma lesion. By using the image processing technique, Alcon et al. [19] give a method for the detection of skin lesions. This method uses the medical history of the patient before the diagnosis begins. Cavalcanti et al. [20] presented a method for automatic skin cancer detection using the fractionation step and feature extraction step. To deal with the increase in false alarms, a modified two-level skin retest classifier was introduced, which labeled the lesion as benign. The amount of medical and biological data being collected is continually increasing. Artificial intelligence and machine learning methods are becoming increasingly popular for analyzing such large and complicated data. As a result, deploying new strategies for recognizing medical and biological characteristics is critical. Especially, deep learning techniques have been widely used to interpret imaging data [21,22]. Skin cancer is among the most difficult health conditions to treat. Several AI-based skin cancer prediction approaches have been employed by several researchers. The segmentation of skin lesions using deep learning techniques is suggested when employing the proposed FrCN approach, with full-resolution attributes learned of each pixel of input data [23], which authors have described as a unique technique for detecting melanoma skin cancer. To obtain a high-quality image, the system prepossessed the skin lesion input image. For segmentation, threshold and edge detection algorithms are applied. The ABCD rule is used by this technique [11] to fetch important features from the segmented image. These retrieved attributes distinguished between non-melanoma cancer and melanoma skin cancer in the photograph. The study [24] proposed a method for distinguishing between normal and pathological skin. First, the system used a starting value to preprocess the dermoscopy images and the segmentation. To extract features, the gray-level co-occurrence matrix was used, and the principal component analysis was used for the selection of features. The biopsy method [25] is a traditional approach for identifying skin cancer. The biopsy approach involves scraping a portion of the skin lesion and sending it to a laboratory for testing. This procedure is invasive, unpleasant, and lengthy. As a result, computer-assisted diagnosis is employed to detect skin cancer in order to solve the aforementioned obstacle. This [26] technique necessitates the use of a skin picture in order to avoid physical contact with the body. This approach will alleviate discomfort while remaining non-invasive. Image processing methods are used in computer-aided diagnostics for melanoma skin lesion detection [27]. The first stage in this type of method is preprocessing the image, followed by image segmentation, which separates the lesion parts. Using the feature extraction technique, the relative features are extracted from the segmented lesions. Following the feature extraction technique, classification is used to divide the skin image into two categories: normal skin and melanoma skin cancer. To diagnose melanoma, use a pattern recognition algorithm to extricate the region of interest (ROI) from the dermatoscopic pictures. The proposed [28] pattern recognition technique achieves a high level of classification accuracy. It is possible to extract efficient texture and color gradients. A technique for segmenting skin lesions based on saliency in dermatoscopy images is reported in [29]. The approach aids in the detection of melanoma skin lesions in sufferers. However, the numerical complexity of this method is larger. As a result, numerous academics have provided a variety of existing methods; however, the timely identification and detection of melanoma skin cancer at its early stage remains a difficult task. An approach for diagnosing skin cancer based on local and universal traits was proposed by Barata et al. [18] using dermoscopy images from Hospital Pedro Hispano, Matosinhos, obtaining results of 96% for sensitivity and 80% for specificity.

## 3. Proposed Methodology

**Figure 3:**
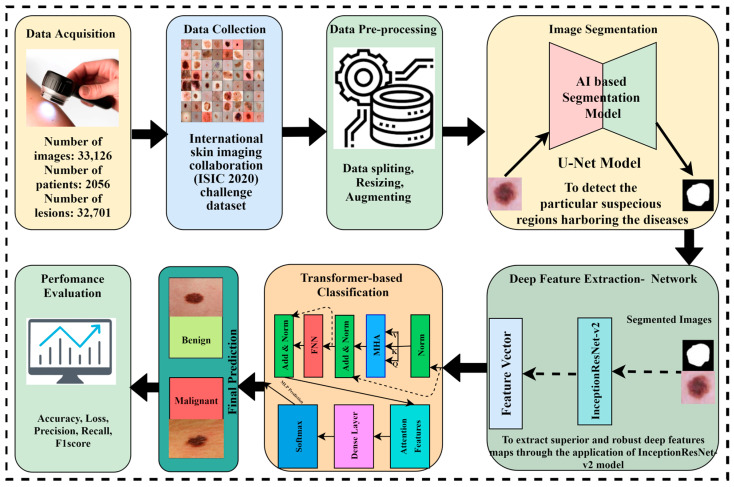
The proposed AI-based framework for skin lesion prediction.

The proposed methodology is based on four major phases. Initially, the dataset was collected from the International Skin Imaging Collaboration (ISIC) and preprocessing techniques were performed on the data. The image segmentation is performed through the U-Net model to obtain targeted segmented masks and to avoid false detections by isolating the relevant areas for analysis. Afterward, the segmented masks and original images are given to the Inception-Resnet-v2 model for optimal extraction of features because Inception-Resnet-v2 has learned rich feature representations for a wide range of images. In the pipeline of the proposed framework, it is important to note that the output feature maps of Inception-Resnet-v2 are flattened to a sequence of patches and become the input of the Vision Transformer, where the self-attention mechanism refines the features to a spatial relationship, and after this transformation, a classification head is applied to receive the desired outcome. The Vision Transformer model with a self-attention mechanism is applied to perform the critical task of skin cancer classification in terms of malignant and benign lesions. Figure 3 depicts the novel proposed framework for skin lesion segmentation, extraction, and classification.

Numerous data manipulations take place during the preprocessing phase of an image. Scaling images, eliminating the background including the k-mean algorithm, the automated computer-aided method, saliency, convolution and deconvolution networks, fuzzy algorithms noise, and enhancing contrast are all instances of such manipulations. The images were then divided as follows: 30% for testing, 10% for validation, and 60% for training. The suggested method utilizes U-Net architecture [30] to both train the model and determine the optimal model hyperparameters. The automatic segmentation of the dermoscopic images after processing using the U-Net model allows for the extraction of regions of interest (ROIs). A deep convolution neural network (CNN), namely Inception-ResNet-v2, is tested and trained on samples of tumor and healthy tissue after the ROIs have been automatically extracted. After that, we used the Vision Transformer, which allows the model to weigh the importance of different elements in the image patches based on their relationship to each other, for the melanoma skin lesion classification.

### 3.1. Dataset

In this work, we have used dermoscopy images from the 2020 skin imaging challenge dataset compiled by the ISIC. Beginning with the dataset ISIC 2020, we preprocessed the data before feeding them to our training model. The ISIC dataset, created for a 2020 challenge to identify melanoma skin cancer, is available on the official site [31]. Notably, 48 GB of huge data is devoted to this dataset to train the model with more variety, complexity, and class balance. There are DICOM image metadata in the collection. This is the standard for medical image data exchange, and it allows for the retrieval of comprehensive patient and image-related data. There are 33,126 dermatoscopic images in the dataset, including both malignant and benign skin lesions. Each photo represents a specific individual and is associated with that person by means of a patient ID.

### 3.2. Data Preprocessing

**Figure 4:**
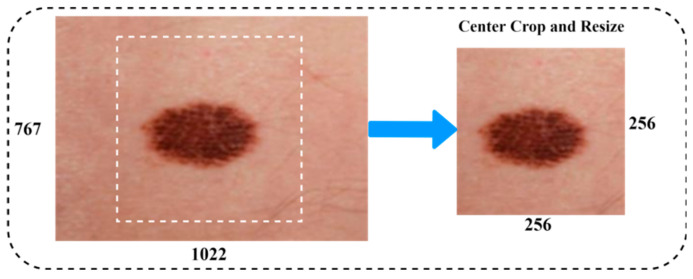
Image resizing.

The aim of this step is to improve image quality by deleting irrelevant parts from the dermatoscopy images before they are processed further. Filters were utilized in the preprocessing of the data to reduce noise and increase the efficiency of the individual components. The procedure for processing the skin lesion image primarily consisted of two stages. Figure 4 illustrates an example of a dermoscopy image after being resized. Data augmentation is commonly used to improve the performance of the classification task on medical image datasets. Data augmentation techniques included a vast range of arbitrary transformations that included horizontal and vertical inversions, scale transformation, rotation transformation, zoom magnification, and brightness to augment the data for training, and almost generated 6562 images to balance the class of malignant scans. The details of the image augmentation methods with settings are as follows: scale transformation with ranges (zero to one), rotation transformation of 25 degrees, zoom magnification of 0.20, horizontal and vertical inversions with true, and arbitrary transformation of 20 degrees. Furthermore, the preprocessing techniques, i.e., centering and resizing the input images, have been utilized to achieve a newly generated image from the original image in the dataset.

### 3.3. Data Splitting

Train and test partitioning evaluate machine learning models. Test and train splitting can be used for classification or regression in supervised learning. Divide the dataset into two subsets. The first is a training dataset for model fitting. Instead of training the model with the other subsection’s dataset inputs, predictions are created and compared to the predicted value. The dataset is split into 30%, 10%, and 60% for testing, validation, and training, respectively.

### 3.4. Image Segmentation

**Figure 5:**
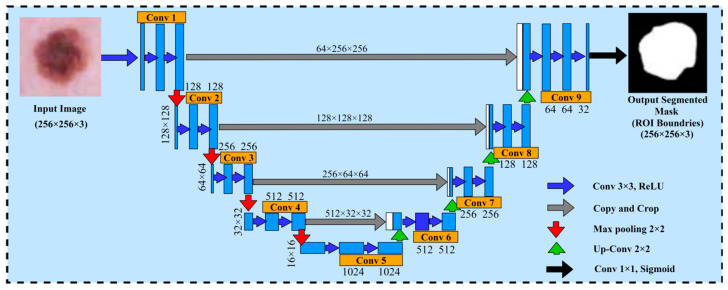
U-Net architecture for segmenting the skin cancer.

The U-Net model is used as a method of segmented medical images and it was first proposed by Ronneberger et al. [30]. The U-Net architecture is composed of two major components, namely the encoder and decoder. The encoder part generates and encodes the more precise maps of high-level features through two-dimensional (2D) convolution and pooling layers, whereas the decoder part regenerates the maps of features to align with the dimensions of the original image demonstrated with spatial resolution. Due to the state-of-the-art results obtained from the U-Net architecture in the domain of computer vision and medical research for image segmentation, we decided to choose the U-Net architecture for the dermoscopic medical image segmentation task in our proposed framework. In the proposed framework, the basic objective of the U-Net model was to identify the affected areas of skin cancer disease. Furthermore, the U-Net model assisted to employ the pairs of images and masks for the purpose of training and the optimization of the loss function. Moreover, to improve the network performance and stability, it uses the 3 × 3 convolutions along with the ReLU activation and batch normalization. However, the max pooling layer is used to decrease the spatial resolution of maps regarding features while training the large input sized images, and is effective in reducing the trainable parameters. This architecture has a reduced overhead compared to other fully connected architectures for capturing context, and a symmetrical extended path enabling automatic border recognition and precise localization with fewer parameters than feed-forward networks. Therefore, this architecture is successful with a dataset of medical images. The core idea is to add a continuous layer to a regular contract network, where the pooling operation is replaced by the up-sampling operator. There is the pixel size and dimension path of the filters for each layer. Within each layer, conv 3 × 3 Relu is the activation function. The max pooling 2D layer is the size of the filter within each layer. Therefore, these layers improve the tendency of the output. After each convolutional layer, the Adam and Adamax optimizer and dropout function provide the loss and accuracies. This architecture comprises a reduced path and an extended path providing a U-shaped architecture. The model architecture is shown in Figure 5.

Tuning hyperparameters is crucial for managing a machine learning model’s behavior. The results confirm that some hyperparameters are relatively more important than others. Therefore, understanding the role of each hyperparameter and its possible effects is an essential skill in deep learning training that makes algorithms more scientific. The hyperparameters are as follows: (i) Learning rate: The learning rate [32] indicates the size of model weight update in the deep learning optimization algorithm. The learning rate constantly and slowly decreases and can be based on adaptation and momentum. (ii) Batch size: In each neural training network, the number of samples sent to the model is known as the batch size [32]. In convolutional neural networks (CNNs), huge batches often cause the network to converge more quickly, but storage resource limitations can lead to inadequate storage if the batches are too large. (iii) Optimizer: Adam [33] is currently a widely used optimizer that converges quickly. (iv) Number of iterations: The number of iterations indicates how many times the neural network was trained using the complete training set. The current number of iterations can be considered reasonable if there is not much of a difference between the test and training error rates. It indicates that there is too much iteration if the test rate first drops and then rises. If it is too large, then you must reduce the number of iterations, otherwise overfitting may occur [32]. (v) Activation function: in a neural network, the activation function does not actually activate anything, but uses the activation function to add nonlinear elements to the neural network, making the network better suited for more complex problems [34]. As the learning algorithms learn, hyperparameters are used, but they are not part of the final model. At the end of the learning process, the machine learning algorithm has the trained model parameter, which efficiently refers to the model. One of the most critical issues while training a machine learning model is overfitting. To mitigate overfitting and increase the efficiency of the trained model, the model should be trained for an optimal number of epochs. Therefore, we trained our model by using a specific number of epochs to get a precise and accurate result.

### 3.5. Feature Extraction

**Figure 6:**
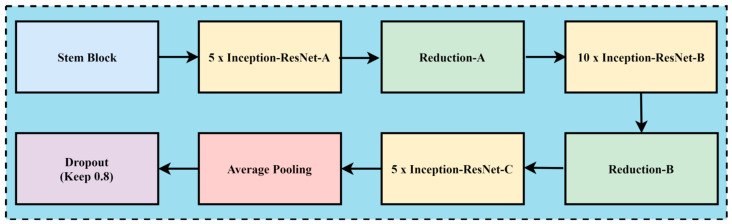
The basic model of Inception-Resnet-v2.

In the case of melanoma prediction through dermoscopic images, the combination of residual connections with Inception-v2 helps to enhance accuracy, better feature retention, enhance generalization with faster training, and reduce diagnostic errors, while without residual connections, the framework might lead towards misdiagnoses due to the complexity of melanoma prediction. The convolution neural network architecture, named Inception Resnet-v2, incorporates residual connections to improve its performance. The Inception family of architectures serves as the foundation for this design, but is enhanced by adding residual connections in place of the Inception architecture’s filter concatenation stage. These networks are types of neural networks that are frequently utilized for image recognition and classification applications. These designs first extract features from an input image using a sequence of convolution layers, and then they perform classification using one or more fully connected layers. Inception-ResNet-v2 was created with high-accuracy image classification in mind, all while requiring minimal computational resources. The residual connections technique is combined with the ideas of the Inception family of architectures to create Inception-ResNet-v2. Each of the Inception modules that make up the architecture has multiple pooling and convolution layers. Inception-ResNet-v2 replaces the Inception architecture’s filter concatenation stage with a residual connection, which is the main distinction between Inception-v3 and Inception-ResNet-v2. Resolving the issue of vanishing gradients helps training and enables the network to learn residual characteristics. Other design features of Inception-ResNet-v2 include utilizing factorized convolutions to lower the number of network parameters and decreasing the input resolution of a module prior to applying convolutions. These design choices help to reduce the computational complexity of the network while maintaining high accuracy on image classification tasks. The architecture has been shown to be very effective for skin cancer image analysis and feature extraction while maintaining a relatively low computational cost. A CNN model called Inception-ResNet-v2 uses residual connections to boost performance. In this approach, the residual connections technique is used to improve training and accuracy on picture categorization challenges. The Inception structure and the residual connection are combined to form the basis of Inception-Resnet-v2. Multiple-sized convolutional filters are merged via residual connections in the Inception-Resnet block. In addition to avoiding the degradation issue brought on by deep structures, using residual connections shortens the training period. Figure 6 shows the basic network architecture of Inception-Resnet-v2 [35]. The details of the hyperparameters used for the fine-tuning of the proposed model are the learning rate, batch size, epochs, momentum, and dropout regularization techniques, with the values of 0.001, 20, 15, 0.99, and 0.5, respectively.

### 3.6. The Proposed Classification Approach

The hybrid AI-based framework for skin cancer prediction, as seen in Figure 3, is built using the beneficial benefits of transfer learning. The multiple-headed Vision Transformer (ViT) is integrated for categorization.

(1) $$Attention(Q,K,V)=Soft\max\left(\frac{Qk^{t}}{\sqrt{d_{k}}}\right)v$$

The Vision Transformer with multi-heads was used to enhance the classification performance to process high dimensional feature vectors through the self-attention mechanism, which permits the model to better distinguish complex patterns in the image patches. ViT’s classification stage uses the generated feature vector as input. Self-attention characteristics lead to exceptional performance and reduced dependence on biases particular to vision [36,37]. In fact, the ViT is selected for its accurate detection of objects based on useful, derived features. The core concept behind ViT is self-attention. It employs weights to show the relative importance of each input data unit in an encoder–decoder configuration. In contrast, CNN models concentrate on pixels with receptive fields. Their remote pixel connections are a problem. The attention mechanism seems like a novel method to get beyond this limitation, eliminating redundancy, lowering false-negative outcomes, and detecting informative portions of input images. The proposed work uses the generated feature vector from the backbone of the Inception-ResNet-v2 network to accept and retrain the ViT for this purpose. The proposed model includes a normalizing layer, a multi-head self-attention (MHA) layer, two dense layers with a classification head, and a regression function i-e Softmax regression function, all of which are included in the proposed transformer encoder for skin cancer classification. In Figure 3, 2D patches are linearly concatenated into a 1D vector for the Inception-ResNet-v2 model, which then passes through a transformer encoder that contains MSA (multi-head self-attention) and MLP blocks. The relationship between each patch and every other patch within a single input sequence is ascertained by the MHA using the dot product kind of attention, which is expressed as follows:

$$MultiHead(Q,K,V)=Concat(head_{1},\ldots ,head_{h})W^{o}$$

where

(2) $$headi=Attention(QW_{i}^{Q},KW_{i}^{K},VW_{i}^{V})$$

In the above equation, value dimensional vectors, query vectors, and key vectors are represented by *V*, *Q*, and *K*, respectively. The variance of the product $Qk^{t}$ is denoted by term $d_{k}$, having a zero mean. Furthermore, the product undergoes normalization through its division by $\sqrt{d_{k}}$. The attention score is extracted from the scaled dot product using the Softmax algorithm. The technique, which provides parallel attention to interpret the entire information of the incoming skin cancer photos, constitutes the core of the ViT module. This model can simultaneously attend to get input from various locations and different representation subspaces to multi-head attention. Using different learned linear projections, multi-head attention increases the keys, values, and queries *h* times in an orderly manner. The computation can be written as follows: where the estimates indices are $W_{i}^{Q}\in R^{d_{\mathrm{model}\;x\;dq}},W_{i}^{K}\in R^{d_{{\mathrm{model}\;x\;d}_{k}}},W_{i}^{V}\in R^{d_{{\mathrm{model}\;x\;d}_{v}}}$, and $W^{o}\in R^{{\mathrm{hd}}_{v}{\;x\;d}_{\mathrm{model}}}$.

The MLP block comprises dropout and normalization layers with a dropout rate of 50%, as well as a non-linear layer with 1024 neurons and Gaussian error linear unit (GeLU) activation.

### 3.7. Performance Evaluation

Model evaluation is the most important step in machine learning. It helps to understand the machine learning’s model performance easily. In our study, we used confusion metrics to evaluate our model’s performance. We used Python for the implementation purpose as Python 3.11 IDE [38] is the most suitable, flexible, and easy-to-use programming language. It is mostly used for many machine learning tasks. It is famous for its rich selection of libraries, especially for machine learning. It is an open-source data analysis tool. The key components of IDE include advanced editing, a code analysis tool, the IPython console, charts, debugger, etc. The model report includes precision, recall, the F-Score, accuracy, sensitivity, and specificity, with formulas motioned in Equations (3)–(8), which represent the performance evaluation of the proposed model. Our prediction system has four possible outcomes in total, as shown in Table 1.

(3) $$Precision=\frac{TP}{FP+TP}$$

(4) $$Recall=\frac{TP}{FN+TP}$$

(5) F-Score=2×Recall×PrecisionRecall+Precision

(6) $$Accuracy=\frac{TN+TP}{FP+FN+TP+TN}$$

(7) Sensitivity=TP/(TP+FN)

(8) Specificity=TN/(TN+FP)

## 4. Experimental Results and Discussion

**Figure 7:**
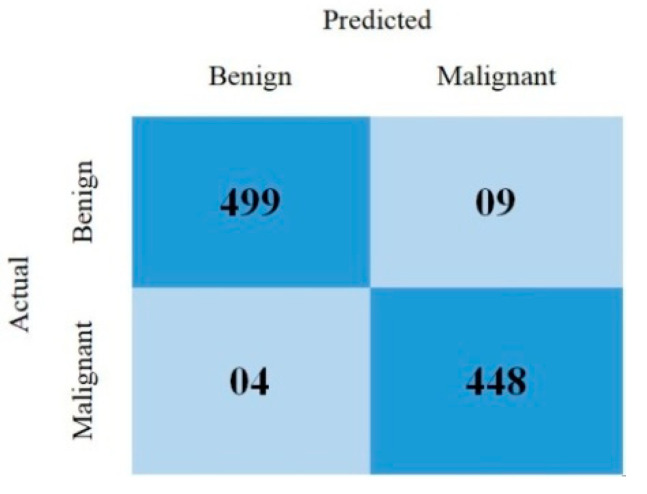
Confusion matrix.

**Figure 8:**
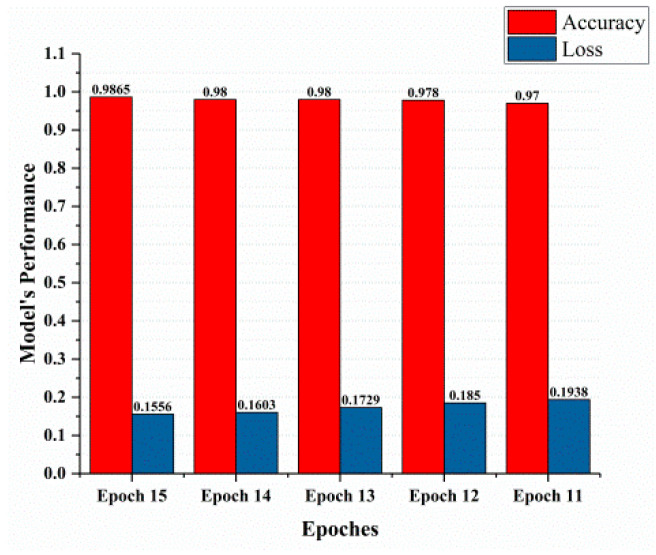
Model’s performance.

**Figure 9:**
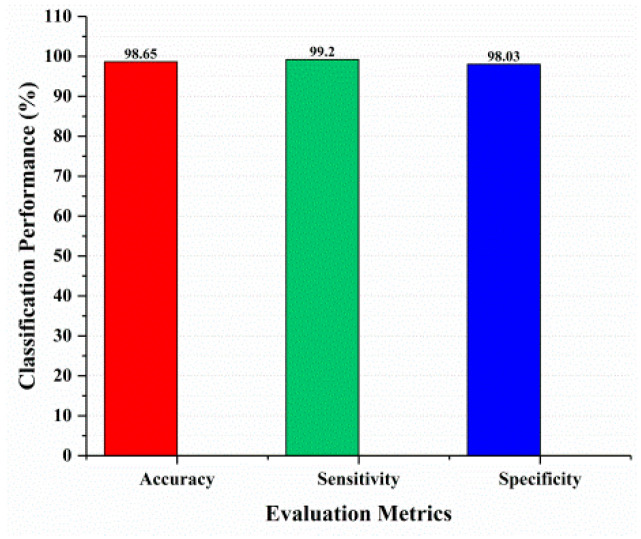
Classification performance.

This section covered the outcomes as we put our work into practice by using a deep learning model involving U-Net architecture for segmentation, Inception-ResNet-2 for feature extraction, and a proposed Vision Transformer for classification. During feature extraction, hyperparameters are tuned until we find the best or optimum values, resulting in the best solution. We have trained our model on 15 epochs and attained an accuracy of 98.65%. The computational resources to train the proposed framework are NVIDIA GeForce RTX 3060 GPU, Intel(R) Core(TM) i7-10700KF CPU @ 3.80 GHz, 64.0 GB of RAM. After training, we plot a confusion matrix for the melanoma and benign skin lesions. True negative, false negative, true positive, and false positive are the foundation of the multi-label confusion matrix (MLCM) [39], which can be expressed as MLCM (0, 0), MLCM (1, 0), MLCM (1, 1), and MLCM (0, 1). In terms of the unique labels y_true and y_pred, multi-label confusion matrices are returned. A confusion matrix is shown for benign and melanoma in Figure 7, while Figure 8 represents the corresponding accuracy and loss of the model against epochs 11 to 15, respectively. The accuracy metric started from 86.03% when the number of epochs was 1, and as it grew, the accuracy increased to 98.65%. Furthermore, Figure 8 presents the model’s loss graph, which shows how badly the model was working. As at the early stage of the model, the number of epochs was lower and at epoch number 1, the loss was high. The proposed model was not doing well at epoch number 1, and while the number of epochs increased, the model’s loss went down and after epoch number 6, the loss decreased and the loss value was 0.3746. We trained our model on 15 epochs and at the 15th epoch, when our model achieved 98.65% accuracy, the loss value was 0.1556, represented by the proposed model’s performance. To mitigate overfitting and increase the efficiency of the trained model, we utilized regularization techniques and optimized hyperparameters to prevent overfitting by adding a penalty term to the model’s loss function. The graphical representation of classification performance in terms of accuracy, sensitivity, and specificity is provided in Figure 9.

From the beginning to the present, great efforts have been made to diagnose melanoma at an early stage in order to save the life of the patient. Artificial intelligence and machine learning methods are becoming increasingly popular for analyzing and helping in the detection of such medical issues. This research uses dermoscopy images from the ISIC 2020 challenge dataset, which is a sizable dataset. Working on a dataset of this size is difficult. We used preprocessing techniques which include image resizing and data augmentation. The approach of this research is not made of a single network, but it is a top-down approach that is subdivided into a series of operations that are needed to perform and achieve the objective of this research work. Therefore, for early and accurate detection of melanoma, we proposed a novel approach combined with U-Net for segmentation, Inception-ResNet-v2 for feature extraction, and AI-based classification with the help of a Vision Transformer. We have trained our model on 15 epochs and attained an accuracy of 98.65%. Model evaluation metrics include loss and accuracy. In model checkpoints, the validation loss value is saved following each epoch. The validation loss value after 12 epochs ends at 0.2773, and the loss value is 0.1556 on 15 epochs. When there is a decrease in the number of epochs, the accuracy loss and validation loss increase. With the increment in the number of epochs, the validation loss and accuracy loss decrease and are met at one point, which is epoch number 12. After epoch number 12, the validation loss and accuracy loss remain at one point, which is 0.1851, and do not increase from this point. Likewise, in model accuracy, as the number of epochs decreases, the validation accuracy and training accuracy reach 95% and 90%, respectively. However, with the increment in the number of epochs, the validation accuracy and training accuracy also increased, and both met at epoch number 6. The suggested methodology’s performance in comparison to previous studies is presented in Table 2.

### Ablation Studies

**Figure 10:**
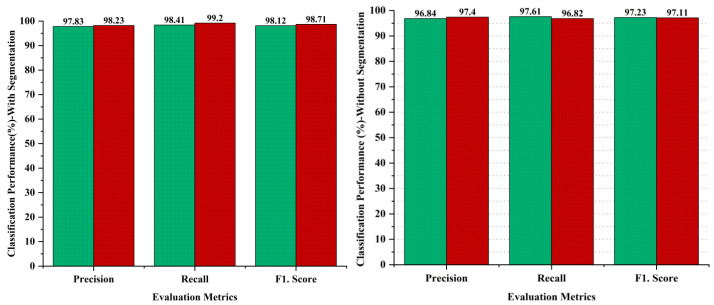
Performance analysis of proposed framework with and without segmentation process; green bar represents benign and red bar represents malignant classes.

Ablation studies are experiments made to identify the significance of a particular part of a complex system by removing or changing it and measuring the impact on the performance of the system as a whole. The aim of the ablative analysis is to identify the better classification by analyzing the iteration-based performance of the models for computing the performance metrics. Ablation studies were also conducted as part of the methodology to evaluate the impact of different components of the proposed system on the overall performance. In the ablation studies, we have highlighted the importance of the image segmentation task, as the image segmentation is performed through the U-Net model to obtain targeted segmented masks to avoid false detections by isolating the relevant areas for analysis. On the other hand, we skipped the image segmentation task in the AI-based framework to perform the ablation experiments, and obtained lower results as compared to our proposed hybrid deep learning approach with the segmentation task. The comparative analysis has been performed to recognize the impact of the segmentation step performed in the proposed approach. The evaluation of the proposed approach with and without segmentation has been provided in Figure 10. The outcome shows that the segmentation process had a significant impact on the diagnosis of melanoma skin cancer, with better results. The comparative analysis has been performed using evaluation metrics, namely precision, recall, and the F1 score to separately judge the performance on the binary classification of malignant or benign. The results indicate that the segmentation process enhanced the performance in the case of benign lesions, recording 0.99% in precision, 0.80% in recall, and 0.89% in the F1.score, whereas 0.83% improved in precision, 2.38% in recall, and 1.6% in the F1.score regarding malignant diagnosis, as shown in Figure 10.

**Figure 11:**
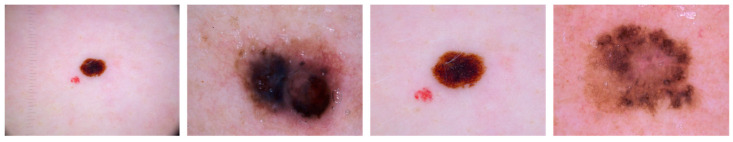
Sample images of melanoma taken from HAM10000 dataset.

To further evaluate the performance of the proposed approach for the prediction of skin cancer, we used a relevant publicly available dataset, HAM 10,000 [52], in the ablation studies. This dataset of 10,015 dermoscopic images is divided into seven categories: basal cell carcinoma (BCC) with 514 images, Dermatofibroma (DF) with 115 images, Actinic Keratosis (AKIEC) with 327 images, Benign Keratosis (BKL) with 1099 images, nevi (NV) with 6705 images, Vascular Skin (VASC) with 142 images, and melanoma (MEL) with 1113 images. We have considered MEL images to test the performance of the proposed approach. The samples of the melanoma images are shown in Figure 11. The performance of the proposed approach on unseen testing data taken from the HAM10000 dataset is almost consistent, given the 98.44% accuracy obtained, indicating that the proposed system can be applied to datasets of varying sizes without significant impact on performance.

## 5. Conclusions and Future Work

A crucial and challenging task in the area of medical research is accurately diagnosing melanoma skin cancer. The main objective of this study is to find a solution for the misdiagnosis of melanoma with the benign lesion, which is a non-cancerous skin lesion which exhibits similar symptoms to melanoma. The proposed hybrid deep learning approach provided better classification results to distinguish the benign and malignant in the case of skin cancer lesions, and achieved an accuracy of 98.65%, 99.20% sensitivity, and 98.03% specificity on the ISIC 2020 challenge dataset, and obtained 98.44% accuracy on unseen data. For future work, we plan to develop a smartphone application and integrate it with our trained model to provide assistance in the early and accurate diagnosis of melanoma.

## Figures and Tables

**Table 1:** Possibilities of predictions.

Cases	Actual	Predicted
*TN*	Negative	Negative
*TP*	Positive	Positive
*FN*	Positive	Negative
*FP*	Negative	Positive

**Table 2:** Comparative analysis of the existing studies and proposed methodology.

Year	Authors	Method/Approach	Accuracy (%)
2018	Al-Masni, et al. [23]	Skin lesion segmentation via deep full resolution convolutional networks	90.78
2020	Kassem, et al. [40]	Deep convolution neural network and transfer learning	94.92
2020	Mousannif, et al. [41]	Convolutional neural networks	86
2021	D. Coronado-Gutiérrez, et al. [42]	DCNN model	85.9
2021	K. Duggani and M. K. Nath [43]	Deep convolution neural network (DCNN) and you only look once (YOLO)	97.49
2022	A. Imran, et al. [44]	VGG, CapsNet, and ResNet	93.5
2022	W. Gouda, et al. [45]	Resnet50, InceptionV3, and Inception Resnet	85.7
2023	Patel et al. [46]	CNN	95
2023	Tembhurne [47]	CNN & Contourlet Transform and Local Binary Pattern Histogram	93
2023	Singh et al. [48]	YOLO, L-Fuzzy Logic	98
2024	Rahman et al. [49]	NASNet model	86.73
2024	Gamage et al. [50]	CNN, Resnet50, VGG16, Xception and transfer learning	98.37
2024	Din et al. [51]	LSCS-Net, U-Net	98.62
Proposed Work		U-Net architecture, Inception-ResNet-v2, Vision Transformer	98.65

## Data Availability

The data used in the experiments are publicly available at “https://challenge2020.isic-archive.com/ (accessed on 15 November 2023)”.

## Abbreviations

AI	Artificial Intelligence
CAD	Computer-Aided Diagnosis
ML	Machine Learning
CNN	Convolutional Neural Network
ROI	Region of Interest
UV	Ultraviolet
FrCN	Full Resolution Convolutional Network
SL	Segmented Lesion
